# Characterisation of a High Fibre Flour Prepared from Soy Milk By-Product and Its Potential Use in White Wheat Bread

**DOI:** 10.3390/foods11233921

**Published:** 2022-12-05

**Authors:** Philip Davy, Timothy Kirkman, Christopher J. Scarlett, Quan Vuong

**Affiliations:** School of Environmental and Life Sciences, College of Engineering, Science and Environment, The University of Newcastle, 10 Chittaway Road, Ourimbah, NSW 2258, Australia

**Keywords:** soymilk by-product, okara, fibre substitute, composite bread, waste utilisation

## Abstract

The commercial production of soy milk renders a large quantity of wet soybean by-product (SMB), which is typically dumped, incinerated, or partially used as animal fodder. This wet SMB has a high moisture content that is rich in nutritional and biologically active compounds. This study aimed to characterise the composition and properties of a flour milled from SMB dried at 100 °C (SMB100) and assess its possible application as a fibre substitute in white bread. The results showed that SMB100 has high levels of dietary fibre (40.6%) and protein (26.5%). It also contains high levels of saponins (31.4 mg/g) and isoflavones (698.0 µg/g). SMB100 has a light-yellow colour with low moisture content and water activity (8.2% and 0.55, respectively). The results also indicated that replacement of wheat flour with SMB100 at 10 or 12.5% by flour weight negatively impacted the raising volume, density, and texture of white bread. Alternatively, substituting wheat flour with 5% of SMB100, did not significantly impact the physical properties of white bread, while significantly improving its dietary fibre content in comparison with the control, revealing that SMB100 is a potential substitute of wheat flour for improvement of dietary fibre in bread. Future studies are needed to optimise bread formulation and improve the processing condition which produces quality white bread with high dietary fibre using SMB100.

## 1. Introduction

Soybean is considered a globally important commodity due to its high nutrient and economic value, which leads to its vast utilisation [1]. Soy milk is widely consumed, and during its commercial production, the water insoluble portion of the soybean cotyledon is removed as a by-product. This wet soy milk by-product (SMB) is often treated as waste, with a predicted global annual production of over 4 million tonnes [2]. Wet SMB, also known by its Japanese name of ‘okara’, contains high levels of nutritional components, including dietary fibre (14.5–58.1%), proteins (24.5–37.5%), and fats (9.3–22.3%) with minor amounts of simple carbohydrates and ash, alongside bioactive compounds, such as isoflavone and soyasaponins [3,4,5,6].

Wet SMB has a moisture content of approximately 85%, leaving it susceptible to rapid microbiological and enzymatic degradation. This contributes to the low utilisation of this industrial by-product, along with other compounding factors. As a result, the majority of wet SMB is dumped in land fills or incinerated, with minor volumes being used as animal feeds [7], thereby creating financial and environmental concerns. With the increasing production of such large volumes of soy milk and other plant based milks, their residues continue to be of increasing concerns. Utilisation of this by-product for human consumption will lower these concerns. With such high nutritional value, coupled with the presence of bioactive compounds, SMB is poised to be a good source of functional ingredients, which can link with various health benefits [8]. In addition, SMB has high concentrations of dietary fibre, which can reduce the glycaemic index of wheat based foods such as bread and noodles [9].

As many compounds in wet SMB are susceptible to oxidation, drying is needed to remove water to reduce degradation, while retaining the nutrient content and phytochemicals in SMB. Our recent study tested different drying conditions and revealed that the most suitable drying condition of SMB is the application of a fan forced (FF) oven at 100 °C. SMB dried by this simple method has an acceptable colour and high levels of bioactive compounds and antioxidant capacity, as compared to vacuum oven and freeze drying, and can be achieved without the need for highly specific drying equipment [10].

The inclusion of fibre in bread is not new to the bread industry, with the availability of additives such as wheat brans, wheat fibre, hi-maize starch and modified starches. Evžen, Miroslava [11] determined that acetylated modified wheat starch can be included in bread at 5% and achieve the best results while increasing the content of fibre through resistant starch. This can also be achieved through the utilisation of products such as SMB, which are often regarded as a waste stream. This not only improves the nutritional properties of a food item but also reduces waste and environmental costs, with potential for financial gain.

Previous studies have incorporated both wet and dry SMB into flour, such as wheat and gluten-free flours, and have utilised SMB to make biscuits and cookies, soy crackers, cakes, sausages, frankfurters, beef patties, noodles, a cheese analogue, vegetable paste and peanut butter [12]. Several attempts have tried to substitute wheat flour with SMB to make bread and have shown the potential application of SMB as a source of dietary fibre [13,14,15,16,17]; however, further studies are needed to validate this practice, especially with regard to the SMB with high fibre content. It is important to characterise the composition of SMB flour prepared from soymilk production and validate its potential use as a wheat substitution in making bread. This study aimed to characterise a flour (SMB100) produced from the previously identified simple drying conditions and further assess its potential application as a fibre substitute in white wheat bread. Direct substitution of this flour into a basic bread dough will highlight the potential benefits, and, furthermore, the limitations, which require more research to overcome.

## 2. Materials and Methods

### 2.1. Materials

Soybeans of medium size and clear hilum were purchased online from Australian Wheatgrass (Riverstone, NSW, Australia). All other food stuffs were purchased from local markets. Analytical and HPLC grade chemicals were purchased from Sigma (Macquarie Park, NSW, Australia): hydrochloric acid (HCl), sodium hydroxide (NaOH), hexane, sodium carbonate (NaCO_3_), calcium carbonate, ethanol (EtOH), methanol (MeOH), sulphuric acid (H_2_SO_4_), phenol, sodium phosphate monobasic, sodium phosphate dibasic, potassium persulfate (K_2_S_2_O_8_), boric acid, methyl red, methylene blue, daidzin, daidzein, genistin, genistein, and total dietary fibre analysis kit.

### 2.2. Methods

#### 2.2.1. SMB Sample Preparation

SMB was produced in the laboratory using a small commercial soy milk machine and followed the process set out by Guimarães, Silva [18], with some modifications. Soybeans were visually inspected, and sub-par beans were removed. Soybeans were then rinsed and soaked in DI water for 12 h at room temperature (RT, 22 ± 1 °C). Water was drained from the beans followed by rinsing with fresh water. Beans were ground with DI water at a dry bean to water ratio of 1:10 (*w*/*v*) in the soy milk machine, followed by pasteurisation of the milk and SMB slurry at 95 °C for 1 min. Soy milk was separated from its by-product through a fine sieve bag, with the remaining soy milk being pressed out by hand. The SMB was laid out on trays and dried in a convection oven at 100 °C as described in our previous study [10], and milled to a flour in a Perten 3100 Laboratory Mill (Perten Instruments, Stockholm, Sweden). The flour (SMB100) was milled through a 0.8 mm mesh, then stored at room temperature, away from light, in a sealed container until required for further analysis.

#### 2.2.2. Proximate Composition Analysis of SMB

Total carbohydrates of SMB were determined using the phenol sulphuric acid method, AOAC Method 44.1.30, described in previous studies [19], with some modifications. In brief, 0.4 g of SMB flour was hydrolysed in 5 mL of 2.5 M HCl for 3 h at 99 °C. Samples were cooled to RT and neutralised with solid NaCO_3_ until effervescence ceased. The volume was made up to 40 mL with DI water, followed by centrifugation at 760× *g* force for 30 min. A 0.05 mL aliquot of the supernatant was added to 0.95 mL of DI water and 0.5 mL of phenol solution (5%), followed by the rapid addition of 2.5 mL of concentrated H_2_SO_4_, to induce a colour change, and allowed to develop for 25 min at 25 °C. The absorbance was measured at 490 nm using a UV–vis spectrophotometer (Varian Australia Pty. Ltd., Mulgrave, VIC, Australia), and results were calculated against a standard curve of D-glucose and reported as grams of glucose equivalents per 100 g of SMB (g GE/100 g).

Total fat content was analysed using the Randall modification of the Soxhlet method [20] and the AOAC method described by Thiex, Anderson [21], with some modifications. Samples of SMB (2 g) were hydrolysed in 4 M HCl using a Velp HU6 hydrolysis system (Velp Scientifica, Usmate Velate, Italy) set at 170 °C for 60 min, followed by filtration and drying under vacuum at 70 °C for 12 h. Fat was then extracted in a Velp solvent extraction unit, SER-148 (Velp Scientifica), using 60 mL of hexane with conditions of 45 min immersion, 45 min washing and 20 min recovery time. The extracted samples were dried in an oven at 105 °C for 1 h to remove excess solvent. The residual fat was weighed, and the final fat content was expressed as grams per 100 g of SMB (g/100 g).

Total protein content was determined using the semi-micro Kjeldahl method with adaption of the AOAC method 960.52 [22]. Sample (1 g) was digested in 20 mL of concentrated H_2_SO_4_ with two copper (ll) catalytic tablets at 430 °C for 2 h in a Velp DKL 12 automatic digestion unit (Velp Scientifica). Digestion was cooled and diluted to 100 mL. For distillation, a 25 mL aliquot of digested solution was alkalised with 25 mL of 32% NaOH, before distillation into a 2% boric acid trap with Tashiro’s indicator (0.2% methyl red, 0.1% methylene blue in 96% EtOH *w*/*v*). Titration of the distillate was carried out with 0.02 M HCl till end point colour change was observed, with results adjusted for a reagent blank [23]. Protein content was calculated based on total nitrogen, with a conversion factor of 5.7 as suggested by Mosse [24], and was expressed as grams per 100 g of SMB (g/100 g).

The total dietary fibre (TDF), soluble fibre (SF) and insoluble fibre (IF) were determined using enzymatic gravimetric methods, AOAC 991.43 and AACC 32–07.01, described by others [25,26], with modifications. SMB100 was defatted with hexane (25:1 mL/g) in an ultrasonic bath set at 100 W, 25 °C for 60 min. The mixture was centrifuged, then the solvent was removed. The defatted flour was dried overnight in a vacuum oven at 70 °C. For enzymatic treatment, 1 g of defatted SMB100 was dispersed in a 40 mL aliquot of 0.08 M phosphate buffer, at pH 6.0, followed by the addition of 50 µL heat stable alpha-amylase and incubated at 95–100 °C for 35 min in a shaking water bath. Mixture was cooled to 60 °C before addition of 100 µL protease and incubation at 60 °C agitated in water bath for 35 min. The pH of the solution was then adjusted to 4.1–4.8 using NaOH or HCl, followed by the addition of 200 µL glucoamylase and application and shaken in a water bath at 60 °C for 30 min. The residue which contains the IF was filtered and washed with 4 volumes of 95% EtOH at 60 °C and SF was allowed to precipitate at RT overnight. Precipitate was further filtered and washed with subsequent treatments of 78% EtOH, 95% EtOH and acetone. Precipitates and residues were dried to constant mass at 70 °C in a vacuum oven. Protein content was determined as previously described, and ash content was analysed using a muffle furnace (LT 5/12, Nabertherm, Lilienthal, Germany) at 550 °C. The SF and IF content were finally calculated by deduction of protein and ash content, and reagent blanks. The values were expressed as grams per 100 g of SMB (g/100 g).

#### 2.2.3. Determination of Phytochemicals

##### Extraction of Phytochemicals from SMB100

SMB100 (0.25 g) was extracted in 10 mL of 50% MeOH using an ultrasonic bath (Soniclean, 220 V, 50 Hz and 250 W, Soniclean Pty Ltd., Thebarton, Australia) set at 30 °C, with 150 W for 1 h with agitation once every 5 min. The extract was then centrifuged at 760× *g* force for 20 min and then stored at −18 °C for further determination of the total phenolic, saponin and isoflavone content.

##### Total Phenolic Content (TPC)

The method described by Vuong, Hirun [27] was used with some modifications. In brief, a 1 mL aliquot of the extract was added to a test tube with 5 mL of 10% Folins–Ciocalteu reagent, mixed via vortex and rested for 8 min. A 4 mL aliquot of 7.5% NaCO_3_ solution was added, re-vortexed and allowed to incubate for 1 hr at RT in a darkroom covered with foil. Absorbance was tested at 765 nm, baseline set by reagent control in a UV–vis spectrophotometer (Varian Australia Pty. Ltd., Mulgrave, VIC, Australia), with results compared to a gallic acid standard curve and reported as milligrams gallic acid equivalents mg/g (GAE mg/g).

##### Total Saponins (TS)

The concentration of saponins in flour was assessed by the process set out by Hiai, Oura [28]. A 0.5 mL aliquot of diluted sample and 0.5 mL of 8% (*w*/*v*) vanillin in MeOH solution was mixed with 5 mL of 72% H_2_SO_4_. Samples were then incubated in a water bath at 60 °C for 1 h, cooled on ice to room temperature before measurement of the absorbance of solutions at 560 nm. Escin was used as the standard, with the results expressed as mg of escin equivalents per gram by dry weight (ESE mg/g).

##### HPLC for Genistin, Diadzin, Daidzein and Genistein

Up to 12 different isoflavones have been isolated from soybean, with the same being possible from SMB [29]. This study aimed to quantify genistin, diadzin, and their aglycones, daidzein and genistein, using HPLC with a photodiode array detector (Shimadzu 20 series, UV–Vis detector) as described Montero, Günther [30], with modifications. In brief, the sample extracts were filtered through 0.2 µm syringe filter into HPLC vials. A reverse phase HPLC column (Agilent Raptor C18 column 4.6 × 150 mm with 5 µm particles (Restek Corporation, Bellefonte, PA, USA)) was used under column temperature of 30 °C, and auto-injection was applied with a 20µL injection volume. Mobile phase consisted of 0.2% formic acid solution (solvent A) and 100% acetonitrile (solvent B), with a solvent flow rate of 1 mL/min analysed at 254 nm. A solvent gradient was developed, with increasing concentration of solvent B as follows: 5 min 0% of solvent B increasing to 25% by 15 min, 40% after 25 min, 60% by 35 min, decreasing to 10% at 40 min and 0% at 45 min. Standard curves were developed for all isoflavones using the same procedure, from 500 µL/mL to 31.25 µL/mL, with retention time of 18.7 min, 19.8 min, 22.8 min and 26.0 min, for daidzin, genistin, daidzein and genistein respectively.

#### 2.2.4. Physical Properties of SMB100 Flour

The moisture content of the flour was determined by mass difference following drying using a fan forced laboratory oven (LABEC Laboratory Equipment Pty Ltd., Marrickville, NSW, Australia.) at 105 °C until constant weight. Water activity was analysed by Aqualab Pawkit water activity meter (METER ^®^, Pullman, WA, USA). The bulk density (ρB), the tapped bulk density (ρT), Carr’s compressibility index (CI) and Hausner ratio (HR) were determined as described by Barretto, Buenavista [31], CI% = (ρT − ρB) × 100/ρT and HR = ρT/ρB.

The water solubility index (WSI) was measured following Hang et al. (2020) [32]. The water solubility index was calculated as WSI = (m_1_ − m_2_) × 100/m_1_, where m_1_ is the initial sample mass of powder and m_2_ is the mass of the dried supernatant. The swelling capacity (SC), water holding capacity (WHC), water absorbance capacity (WAC) and oil binding capacity (OBC) were determined according to a previous study [33] to determine the effects of water and oil on the functional attributes of SMB100 at RT. The CIELab colour space with factors of L*, a*, b* were determined using a hand-held Minolta Chroma Meter (CR-400 Chroma meter, Konica Minolta Sensing, Sakai, Osaka, Japan). These values were then transformed into hue angle (H = tan^−1^ (b/a)) and chroma (C = $\sqrt{a^{2}+b^{2}}$) which describes colour and saturation [34].

#### 2.2.5. Bread Dough Formulation and Baking

Bread dough formulation and baking methods were developed based on previous studies [17,35], with modifications. Formulations of control and substitution with 5%, 10% and 12.5% of flour with SMB100 are shown in Table 1.

To form the dough, all ingredients were added to a planetary mixer fitted with a dough hook and allowed to form a homogenous mixture for 2 min at low speed. The speed then increased to approximately 120 rpm to rapidly promote gluten formation and was sustained for 13 min. Dough was placed in an air tight container and allowed to ferment at 40 °C for 90 min, with a fermentation schedule of 20 min, first punch, 20 min, second punch, followed by moulding, tinning and a final proofing for 50 min. Loaves were then baked at 200 °C for 22 min, cooled, and assessed the following day for mass, volume, relative density, colour and textural profile.

#### 2.2.6. Volume, Relative Density, and Texture Profile Analysis (TPA) of SMB Bread

All loaves were weighed on a top loader balance, with the mass recoded, before measurement of volume by the rapeseed displacement method [36]. The relative density of the loaves was determined as the mass/volume (g/mL). Texture profile of crumb was analysed using a Shimadzu Texture Analyser EZ-LX (Shimadzu Scientific Instruments, Pty. Ltd., Rydalmere, NSW, Australia) according to method described by Zambelli, Galvao [37], with modifications. Bread was cut into 20 mm slices and samples (*n* = 3) of each loaf were stored in an airtight container until tested. A 5 N load cell fitted with a 12.5 mm cylinder probe, with a cross head speed of 10 mm/sec, was used, with a two bite, 50% compression (10 mm), textural profile analysis (TPA) to measure hardness (N), chewiness (N) and cohesiveness (%) using Trapezium X ^©^ material testing operation software.

#### 2.2.7. Colour of the Crust and Crumb

Hue angle (H°) and chroma (C) of crust and crumb were determined as discussed above. The change in colour from the control loaf was determined by total colour change (ΔE = $\sqrt{\Delta L^{2}+\Delta a^{2}+\Delta b^{2}})$, total saturation change (ΔC = $\sqrt{\left(a^{2}\mathrm{sample}+b^{2}\mathrm{sample}\right)}-\sqrt{\left(a^{2}\mathrm{control}+b^{2}\mathrm{control}\right)}$), and the total hue difference (ΔH = $\sqrt{{\left(\Delta E\right)}^{2}-{\left(\Delta L\right)}^{2}-{\left(\Delta C\right)}^{2}}$) [38].

### 2.3. Statistical Analysis

All experiments were performed in at least triplicates and results were determined as the means ± standard deviations (SD). One-way ANOVA (analysis of variation) and all pairs Tukey HSD were used to compare the means and to determine the connecting letters report using JMP^®^ Pro statistical software 14.0.0 (SAS Institute Inc., Cary, NC, USA) with a statistically significant level of *p* < 0.05.

## 3. Results and Discussion

### 3.1. Quality of SMB100 Flour

#### 3.1.1. Proximate Composition of SMB100 Flour

The wet SMB before drying had a moisture content of 83.4 ± 0.3%. After drying and grinding through 0.8 mm mesh, the nutrient content, phytochemicals, and physical properties of SMB100 flour are shown in Table 2. Overall, SMB100 had approximately 26.5% protein, 14% fat, 23% carbohydrates, 4% ash, while insoluble fibre accounted for 38.4% and soluble fibre for 2.2%. These findings are in line with previous results, which found soybean residue has high levels of fibre and protein [33]. In comparison to wheat flour and dehulled soy flour, SMB100 had higher protein content than that of wheat flour (14.7%), but much lower than that of dehulled soy flour (49.3%). SMB100 had 86% higher fat content than that of wheat flour, but 44% less than dehulled soy flour. SMB100 had lower carbohydrate content than wheat flour (72.7%), but higher than dehulled soy flour (18.6%). SMB100 had a higher level of ash in comparison to that of both wheat flour and dehulled soy flour. Of note, SMB had a significantly higher level of dietary fibre in comparison with that of both wheat flour and dehulled soy flour. Dietary fibre in SMB100 was 48-fold and 13-fold higher than that in wheat flour and dehulled soy flour, respectively [39,40]. Our findings revealed that SMB100 flour has great potential for use as a food ingredient, due to its high levels of dietary fibre and protein.

SMB100 was shown to have high levels of phytochemicals (Table 2). SMB100 had a total phenolic content of 8.2 mg/g and a total saponin content of 31.4 mg/g. For the major isoflavones, SMB100 had approximately 339 µg/g of genistin and 268 µg/g of daidzin. The aglycones forms of these isoflavones were 44 µg/g daidzein and 28 µg/g of genistein. This results in a total assayed isoflavone content of 698 µg/g. The lower content of aglycones, compared to the glycated forms, has been noted in other studies [41,42]. Total phenolic content in SMB100 was lower than that in the soybean of 24 genotypes (24 mg/g) [43] However, saponins and isoflavones were comparable with those in soybean [44]. The difference can be explained by several influencing factors, including cultivar and processing conditions [45,46]. High levels of phytochemicals are advantageous for use of SMB100 in further applications when compared to other flours, such as wheat or corn, as they are linked to improving human health [47].

The presence of these components, particularly the presence of isoflavones, demonstrate the benefits of utilising this by-product for human consumption rather than the current methods of disposal. This is further true when considering the current adjuncts used to fortify dietary staples such as breads. While using modified starches provides fibre addition with tailored rheological properties, they require intervention to produce these components, with limited or no other nutritional benefit. Reports suggest that isoflavones can act as antioxidants to retard the oxidation of fats, provide benefits to cardiovascular health and some cancers, and act as an estrogen supplement for post-menopausal women [48].

#### 3.1.2. Physical Properties of SMB100 Flour

The physical properties of the milled flour were studied to highlight the bulk storage and distribution capacity, interactions with water and oil, and a qualitative description of colour (Table 2). After milling, SMB100 flour has a moisture content of 8.6% and a water activity of 0.55. Due to its low moisture content and water activity, SMB100 flour can be considered a shelf-stable commodity during storage and transportation [49]. Bulk density and tapped bulk density of SMB100 flour were 0.24 g/mL and 0.44 g/mL, respectively. When comparing the tapped and levelled bulk densities for compression, the Carr’s CI showed a 44.65% compressibility and HR of 1.81. Densities and resulting CI and HR can be used to highlight the storage capacity, tendency to compress, and flowability of a powder. Barretto, Buenavista [32] have reported a rating scale for powders flow characteristics, with an excellent flow determined to have a CI of ≤10% or HR ≤ 1.11, through to a very, very poor powder, with a CI ≥ 38% or HR ≥ 1.60. As such, under this scale, SMB100 flour has been characterised as having a very, very poor flowability.

The ability of a powder to interact with liquids can highlight functional characteristics for further application. The ability to hold water can influence the textural qualities of a food product, which can lead to both advantages and limitations. In bread, additions which are high in fibre can affect the rheological properties of both dough and baked loaf. During dough formation, fibre can absorb water and thus result in incomplete hydration of glutenous proteins, which leads to lower loaf volume and a firmer crumb texture. Therefore, the ability of flour to hold water can improve the shelf life of baked bread and slow the action of syneresis [50]. When SMB100 flour was exposed to excess water, a swelling capacity of 6.1 mL/g, water holding capacity of 9.9 mL/g, water absorbance capacity of 7.8 mL/g, and a water solubility index of 12.7% was observed. These results are in agreeance with the results of Ostermann-Porcel, Rinaldoni [33], who compared multiple drying conditions for okara. Similar results were observed for oil binding capacity, with results of 3.2 mL/g. In addition, SMB100 has a lightness (L) of 89.3, hue angle of 83.3 ± 0.08 and chroma saturation of 16.9 ± 0.3, showing a mild yellow colour [51]. Considering these results, it could be inferred that SMB100 has a high capacity to absorb and hold water, with limited ability to dissolve into a solution. In products such as bread and other dough products, this will aid in retaining moisture in the final product, however fibre may steal moisture away from another component. This can be overcome by increasing the moisture content to compensate for this action. Having the ability to hold both water and oil may have advantages when developing food emulsions.

### 3.2. Substitution of Wheat Flour with SMB100 in Production of White Wheat Bread

#### 3.2.1. Impact of SMB100 Flour Substitution on Loaf Mass, Volume, Relative Density and of White Bread

During this study, bread was successfully formulated with the addition of SMB100 flour with varying results. With an aim of increasing the fibre content in bread while utilising an industry by-product, bread was formulated with SMB 100. The increase in fibre content was determined from the ingredients used. SMB 5% had a calculated fibre content of 26.6 g/loaf, while SMB 10% and SMB 12.5% had levels of 35.7 and 40.3 g/loaf respectively. This is an increase from the standard formulation (control), which only had 17.5 g of fibre per loaf. The WHO has recommended that an adult diet contains greater than 25 g of dietary fibre per day. A serving (two slices) of SMB 5% would contribute 3.3 g of dietary fibre while 10% SMB would contribute 4.4 g/serving, which equates to 12.7% and 16.9% of the recommended daily intake of fibre [52]. Further, Food Standards Australian New Zealand (FSANZ) have denoted that to be classed as a high source of fibre, bread must contain at least 4 g of fibre per serving [53]. Based on this evidence, there would be greater merit to formulating bread with 10% or higher of SMB100 flour.

**Figure 1:**
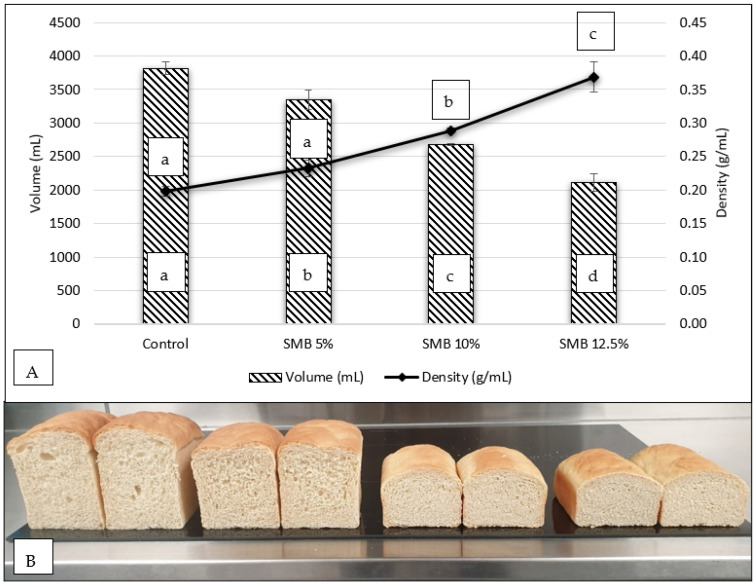
(**A**) Substitution of SMB100 flour in white bread, formulated with 0%, 5%, 10% and 12.5% and its impact on volume, relative density, and water activity. (**B**) White wheat bread loaves formulated with SMB100 flour (left-right), control (0%), 5%, 10% and 12.5%. Results are means ± standard deviations. Results from same test not sharing similar letters are statistically different (*p* < 0.05).

The impact of SMB100 flour substitution on volume and relative density of white bread is presented in Figure 1. After baking and cooling for 24 h, the loaves exhibited no statistical difference (*p* > 0.05) in their mass, with a range of 754–773.8 g. The inclusion of SMB100 flour did significantly (*p* < 0.05) influence the volume of all loaves, with the largest volume from control loaves at 3816.7 mL. From the results (Figure 1A) the volume of each treatment was reduced as substitution level increased. Bread formulated with 5% SMB100 flour had a displacement volume of 3347.5 mL, while 10% and 12.5% had volumes of 2680 mL and 2106.7 mL. This decrease in loaf volume can be observed in Figure 1B, which shows examples of white bread loaves produced for this study. The relative density of bread is determined from a ratio of mass to volume, with an increase in density related to a heavier crumb texture. From the results (Figure 1A), the control and SMB 5% had no statistical difference (*p* > 0.05) in loaf density, with 0.20 and 0.23 g/mL, respectively. Both SMB 10% and 12.5% had significantly higher densities than that of control and SMB 5%. A reduction in volume in combination with an increase in density has been reported in other studies where composite flours were integrated with wheat flour in bread [54]. Considering these results, it can be suggested that SMB100 flour can be substituted into standard white bread formulation at a dose rate of 5% without effecting relative density, however a reduction in volume may be observed.

**Figure 2:**
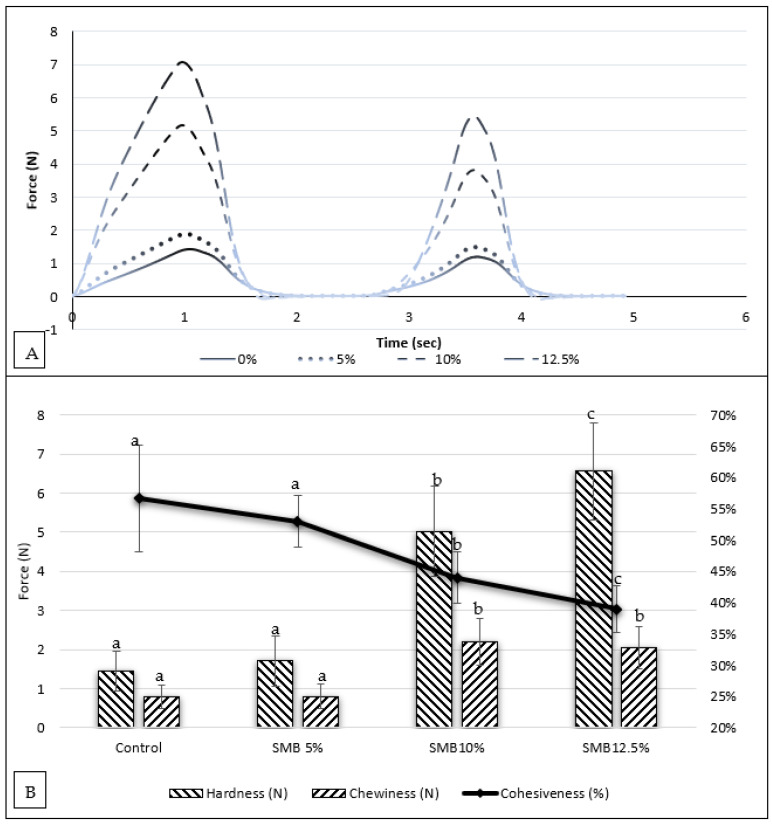
(**A**) Textural profile analysis graph (Force (N)/Time (second)) of white bread formulated with 0%, 5%, 10% and 12.5% SMB100 flour. (**B**) Textural results for hardness (N), chewiness (N) and cohesiveness (%). Results are presented as mean (*n* = 9) ± SD, with different connecting letters for the same test are considered statistically different (*p* < 0.05).

To further determine the effects of inclusion of SMB100 in white bread on the forces that are required to bite and chew a slice of white bread, a textural profile analysis was undertaken. The textural profile is shown in Figure 2A.

Hardness is the maximum force (peak 1 max (N)) required to deform a sample during the first compression cycle. The results (Figure 2B) showed that white bread was firmer with increasing level of SMB100 substitution from 5 to 12.5%. Cohesiveness is a measure of the interaction of the food matrix to adhere to itself during compressions and is determined from the quotient of compression 2 to compression 1 from the area under each curve. The cohesiveness of the crumb (Figure 2B) for the control and SMB 5% were not considered to be different (*p* > 0.05) at 57% and 53%, respectively. While loaves formulated with 10% and 12.5% had cohesiveness of 44% and 39%, respectively, which were statistically different from other measurements. Chewiness has a relationship with both hardness and cohesiveness. Chewiness increased statistically once inclusion reached 10% and higher. From the textural characteristics of bread substituted with SMB100, it can be asserted that as the inclusion rate increases, the hardness, cohesiveness, and chewiness will be affected in a negative manner. At an inclusion rate of 5%, it is possible to produce bread that is similar in textural qualities to the control. Noticeably, substitution of SMB can be increased, but the density and texture may not be accepted by the consumers, as a recent study found that bread made of wheat, millet and SMB has low hedonic score [55]. Therefore, future studies are needed to test the best level of substitution of flour for SMB when dried under different conditions.

Changes in volume, density and textural properties of bread formulated with composite flours has been reported to result from a reduction in gluten due to the substitution. During mixing of the dough for the current study, it was noted that as substitution increased, the dough became increasingly dryer. As SMB100 flour has a high proportion of fibre, it was inferred that this component absorbed moisture. This has been observed by others [56]. As such, there may be insufficient water to enable the complete hydration of glutenous proteins during dough formation and reduced mechanical stretching of the dough. This may have led to incomplete formation of gluten, limiting the elasticity, extensibility, and the final structural matrix of the bread, leading to changes in physical characteristics. Wickramarathna and Arampath [17] attempted to substitute with 10% of SMB, however the fibre content of the by-product in their study was 6.66 g/100 g, which is much lower than that in the flour prepared by this study and this should be considered when using this substitution in making bread, because fibre has strong impact on the texture and density of bread. To increase the substitution of SMB with high fibre content, future studies might consider adding dough improvers or gluten as suggested by Lu, Li [14] and Silva, Paucar-Menacho [18]. The later also increased dough hydration with increases in substitution, something that was controlled for in the present study. Though it is difficult to compare the simple substitution used in the present study with that of others, together, these results further strengthen the assertion that SMB100 can be effectively included in white bread.

#### 3.2.2. Impact of SMB100 Flour Substitution on Colour of Crust and Crumb of White Bread

Colour is an important characteristic of any food item. The inclusion of SMB100 flour had various effects on the colour of baked bread loaves during this study. The results (Table 3) show that lightness of crust increases as more SMB100 flour is included, however SMB 12.5% alone was significantly different (*p* < 0.05) from control and SMB 5%. There were no statistical differences (*p* > 0.05) in hue angle and chroma of crust, revealing the colour is similar across the treatments. To highlight any changes in colour and saturation for crust colour, compared to control loaves, ΔE, ΔC, ΔH° were assessed. For ΔE and ΔH°, the total colour change and change in hue showed the same trend. From Table 3, both SMB 5% and 10% were not statistically different from control, with total colour changes values of 1.62 and 4.85, respectively. SMB 12.5% was different compared to SMB 5% but similar with SMB 10%. When comparing ΔH° for crust colour, the change from control for SMB 5% was 0.42 and 0.70 for 10%, which were statistically similar (*p* > 0.05). Bread with 12.5% substitution rates had the largest shift from control with 1.34, which was significantly different from SMB 5% alone. There was no significant difference between the change in chroma for any treatment for crust colour. Overall SMB100 flour had minimal effect on the colour of the crust of white bread. Lightness (L*) was the only measure that was affected, which is considered in the calculations for ΔE and ΔH°. It may be possible to suggest that the production of higher coloured Maillard reaction end products were retarded during baking.

As observed in crumb colour, the internal crumb lightness (L*) showed an increasing trend as inclusion of SMB100 flour increased. From the results (Table 3), statistical similarity (*p* > 0.05) was shown for control (65.2), SMB 5% (65.2) and SMB 10% (68.9), while SMB 12.5% (70.8) was reported to be different (*p* < 0.05) from control and SMB 5% but shared similarity with SMB 10%. The H° for all loaves were affected by inclusion, with statistical differences (*p* < 0.05) for the mean of each treatment. The angle changed from 87.2 for control to 81.3 for 12.5%, which shows a shift towards the red spectrum as more flour was substituted. The intensity of the colours also changed in a similar manner, with values for chroma changing from the control at 14.5 through to 19.7 for SMB 10%, with all treatments significantly different (*p* < 0.05) from control. When comparing the shift of total colour, hue angle and saturation, SMB 5% showed the least change from control, with results of 2.79 for ΔE, 2.62 for ΔH°, and 0.75 for ΔC. Overall, the results for SMB 10% and 12.5% were statistically independent (*p* > 0.05) from SMB 5%, except for ΔC for SMB 12.5%, which shared similarities with both treatments.

As SMB100 flour has a mild yellow colour, the inclusion of SMB100 flour can have an impact on the colour of the crumb of white bread. Unlike the outer crust of bread, which experiences high temperature, leading to the formation of crust colour, the internal crumb retains moisture, retarding this change in colour, and, as such, there was minimal change in lightness, but a general change in hue angle. This trend in colour changes was not observed by others [17,57].

## 4. Conclusions

It can be concluded that SMB dried in a fan forced oven at 100 °C can be successfully milled to a flour for applications in food as a nutrient substitute, especially for improvement of protein and fibre content. In particular, the physicochemical profile showed high proportions of dietary fibre (38.4% IF and 2.2% SF) and proteins (26.5%), highlighting the nutritional value of SMB100 flour. The low water activity (0.55) indicates a shelf stable powder, which, along with a capacity to absorb water with limited solubility, makes SMB100 a very promising wheat flour substitute.

When incorporated as a white wheat flour replacement in bread, physical properties were affected as substitution levels increased. This was particularly evident in SMB 10% and 12.5%, where loaf volume, relative density and texture profile analysis were negatively affected. However, bread formulated with 5% SMB100 flour exhibited minimal influence on all physical properties tested. Changes in colour of bread were also observed; however, this was most evident in variation of crumb colour. Overall, crust colour was not significantly affected. Considering these results together, it can be asserted that SMB100 can be used as a flour for simple substitute in bread at a rate of 5% (by mass) of white wheat flour. As the test conditions used for this project only considered simple substitution of wheat flour, future studies should be undertaken to optimise the formulation and processing conditions to improve the physical and textural qualities of bread with potentially higher substitution rates. Achieving this would allow the production of a white wheat bread which could be classed as a high fibre food.

## Figures and Tables

**Table 1:** Ingredients used in SMB100 substituted bread. All weights are in grams (g).

Ingredients (g)	Control	5% SMB	10% SMB	12.5% SMB
Flour	500	475	450	437.5
SMB flour	0	25	50	62.5
Water	320	320	320	320
Yeast	5	5	5	5
Oil	15	15	15	15
Sugar	10	10	10	10
Salt	7.5	7.5	7.5	7.5

**Table 2:** Nutrition, phytochemical, and physical properties of SMB100.

Nutritive Components						
Proteins (g/100 g)	Fat (g/100 g)	Carbohydrates (g GE/100 g)	Total Fibre (g/100 g)	Insoluble Fibre (g/100 g)	Soluble Fibre (g/100 g)	Ash (g/100 g)
26.5 ± 0.51	13.8 ± 0.93	23.7 ± 3.2	40.6 ± 0.41	38.4 ± 0.95	2.2 ± 0.95	4.1 ± 0.23
**Phytochemicals**						
TPC (GAE mg/g)	Saponins (ESE mg/100 g)	Daidzin (µg/g)	Genistin (µg/g)	Daidzein (µg/g)	Genistein (µg/g)	
8.23 ± 3.66	31.4 ± 2.80	286.3 ± 62.36	339.0 ± 73.35	44.5 ± 11.24	28.6 ± 1.75	
**Physical properties**						
Moisture (%)	Water activity	Bulk density level (g/mL)	Tap bulk density (g/mL)	CI (%)	HR	Swelling Capacity (mL/g)
8.67 ± 0.44	0.55 ± 0.01	0.24 ± 0.003	0.44 ± 0.01	44.65 ± 0.07	1.81 ± 0.002	6.11 ± 0.15
Water Holding (mL/g)	Water absorbance (mL/g)	Water solubility (%)	Oil binding (mL/g)	Lightness (L*)	Hue (H°)	Chroma (C)
9.92 ± 1.5	7.86 ± 0.36	12.7 ± 1.5	3.16 ± 0.17	89.3 ± 0.17	83.3 ± 0.08	16.9 ± 0.30

Results are mean (*n* = 3) ± standard deviations.

**Table 3:** The inclusion of SMB100 flour in white bread, at 0%, 5%, 10% and 12.5%, and the effects in lightness (L*), hue angle (H°), and chroma (C).

**Crust**	L*	**H°**	**C**	**ΔE**	**ΔC**	**ΔH°**
Control	55.5 ± 3.4 ^a^	64.9 ± 2.4 ^a^	29.6 ± 1.6 ^a^			
SMB 5%	54.7 ± 0.38 ^a^	64.3 ± 0.88 ^a^	30.7 ± 1.3 ^a^	1.62 ± 0.46 ^a^	1.14 ± 1.3 ^a^	0.42 ± 0.14 ^a^
SMB 10%	59.0 ± 0.85 ^ab^	65.9 ± 1.3 ^a^	31.9 ± 3.1 ^a^	4.85 ± 1.2 ^ab^	2.33 ± 3.1 ^a^	0.70 ± 0.32 ^ab^
SMB 12.5%	63.1 ± 3.6 ^b^	65.2 ± 3.3 ^a^	27.3 ± 2.0 ^a^	8.19 ± 3.8 ^b^	−2.34 ± 2.0 ^a^	1.34 ± 0.45 ^b^
**Crumb**	L*	**H°**	**C**	**ΔE**	**ΔC**	**ΔH°**
Control	65.2 ± 2.8 ^a^	87.2 ± 0.32 ^a^	14.5 ± 0.67 ^a^			
SMB 5%	65.2 ± 0.78 ^a^	85.0 ± 0.21 ^b^	17.2 ± 0.41 ^b^	2.79 ± 0.46 ^a^	2.62 ± 0.41 ^a^	0.75 ± 0.07 ^a^
SMB 10%	68.9 ± 1.5 ^ab^	82.3 ± 0.23 ^c^	19.7 ± 0.58 ^c^	6.64 ± 1.2 ^b^	5.19 ± 0.58 ^b^	1.56 ± 0.04 ^b^
SMB 12.5%	70.8 ± 0.55 ^b^	81.3 ± 0.50 ^d^	18.4± 0.75 ^bc^	7.11 ± 0.47 ^b^	3.90 ± 0.75 ^ab^	1.78 ± 0.18 ^b^

Results are means (*n* = 4) ± standard deviations. Results in the same column with different superscript letter are statistically different (*p* < 0.05).

## Data Availability

The data presented in this study are available on request from the corresponding author. The data are not publicly available due to privacy.
